# Immunohistochemical expression of smoothened in periocular basal cell, squamous cell and sebaceous carcinomas

**DOI:** 10.1038/s41598-025-06011-y

**Published:** 2025-06-20

**Authors:** Gustav Stålhammar, Basel Haj Hasan, Krzysztof W. Kotowski, Elin Bohman, Emma Lardner, Alexander Berg Rendahl

**Affiliations:** 1 St. Erik Ophthalmic Pathology Laboratory, St. Erik Eye Hospital, Stockholm, Sweden; 2 Ocular Oncology Service, St. Erik Eye Hospital, Stockholm, Sweden; 3 Division of Eye and Vision, Department of Clinical Neuroscience, Karolinska Institutet, Eugeniavägen 12, Stockholm, 17164 Sweden; 4 Oculoplastic Service, St. Erik Eye Hospital, Stockholm, Sweden; 5 Department of Cancer Biology, Dana-Farber Cancer Institute, Boston, MA USA; 6 Department of Genetics, Harvard Medical School, Blavatnik Institute, Boston, MA USA

**Keywords:** Smoothened, Hedgehog, Basal cell carcinoma, Sebaceous carcinoma, Squamous carcinoma, Head and neck cancer, Skin cancer, Tumour biomarkers, Biomarkers, Oncology, Eye cancer

## Abstract

Inhibition of Smoothened (SMO), a key protein in the Hedgehog signaling pathway, is effective for locally advanced basal cell carcinoma (BCC), but is not yet used for sebaceous carcinoma (SEB) or squamous cell carcinoma (SCC). This study quantified SMO expression and its relationship to proliferative activity in non-nodular periocular BCC, SEB and SCC. Tumor samples from 47 patients (17 BCC, 15 SCC, and 15 SEB) were immunostained and analyzed digitally to assess SMO optical density and Ki67 hot-spot index. SMO expression was significantly higher in all tumor types than in surrounding stroma, with no inter-tumor differences. SMO correlated with mitotic count in BCC but not in SCC or SEB, whereas higher SMO consistently paralleled a higher Ki67 index across all three carcinomas. These findings indicate that SMO expression and proliferative activity are closely linked and suggest that Hedgehog inhibitors, proven in BCC, warrant clinical evaluation as adjuvant or neoadjuvant therapy for periocular SEB and SCC.

**Supplementary Information:**

The online version contains supplementary material available at 10.1038/s41598-025-06011-y.

## Introduction

Hedgehog (Hh) signaling is critical for embryonic development but is down-regulated in most adult tissues, with transient activity in certain regions such as the brain, testis, and hair follicles^1^. The pathway is mediated through a cell membrane receptor complex that includes Patched (PTCH1) and Smoothened (SMO)^2^. Aberrant activation of Hh signaling has been implicated in various cancers, particularly basal cell carcinoma (BCC)^2,3^. Selective Hh inhibitors, such as vismodegib and sonidegib, have proven effective in treating locally advanced and metastatic BCC and are now recommended in both adjuvant and neoadjuvant settings^4–8^. The European Association of Dermato-Oncology (EADO) recently introduced a classification system for BCC, categorizing them as either ‘easy-to-treat’ (which encompasses the most common BCC) or ‘difficult-to-treat’ (including all locally advanced BCC). For the latter, Hh inhibitors should be offered^6^.

Sebaceous carcinoma (SEB) is a rare and aggressive cancer predominantly found in the periocular region. It typically presents as painless, subcutaneous nodules and is characterized by invasive growth into the eyelids and conjunctiva^9,10^. Misdiagnosis is common, as SEB can resemble benign conditions such as chalazion in its nodular form or conjunctivitis in its pagetoid spread, often delaying diagnosis by over a year. Pagetoid spread into the skin or conjunctiva significantly increases the risk of orbital exenteration^11^. Despite aggressive treatment, SEB frequently recurs and metastasizes to the cervical lymph nodes^10^. Kaplan-Meier estimates of disease-related mortality 10 years after initial diagnosis are 14% at stage I, 23% at stage II, 61% at stage III, and 100% at stage IV^12^. The extent of surgical margins also affects prognosis, with higher recurrence rates observed in cases with 1–3 mm margins compared to 5 mm margins, though achieving such margins may be challenging, particularly for orbital tumors^13^.

Squamous cell carcinoma (SCC) of the head and neck remains a significant contributor to cancer-related morbidity and mortality, with more than 60 000 new cases diagnosed annually in the United States and 600 000 globally^14^. Previous research has demonstrated that SMO may be expressed in both SEB and SCC, though Hh inhibitors are not yet widely utilized for these cancers^14,15^. In this study, we expand on prior analyses by comparing SMO expression in periocular non-nodular BCC, SEB, and SCC using objective measurements of expression levels with digital image analysis. We also investigate the correlation between SMO expression levels and mitotic count. The observed similarities in expression suggest that Hh inhibitors could be considered as a treatment option for periocular SEB and SCC, similar to their use in BCC.

## Methods

### Study aim

The aim of this study was to evaluate the immunohistochemical expression of Smoothened (SMO) in periocular basal cell carcinoma (BCC), sebaceous carcinoma (SEB), and squamous cell carcinoma (SCC).

### Patients and tumor samples

Formalin-fixed, paraffin-embedded (FFPE) tissue samples were collected from eyelid resections with histopathologically confirmed non-nodular BCC, SEB, and SCC from the archives of the St. Erik Ophthalmic Pathology Laboratory. At least 15 specimens of each tumor type were targeted for collection based on the following inclusion and exclusion criteria:

Inclusion criteria:

1. Excision performed after January 1st, 1995.
2. Histopathologically confirmed diagnosis without ambiguity.

Exclusion criteria:

1. Tissue unavailable in the archive.
2. Tissue smaller than 10 × 10 mm on the glass slide (e.g., core needle biopsies).
3. Tissue unsuitable for immunohistochemical analysis due to artifacts, inflammation, hemorrhage, or necrosis.
4. Written informed consent not obtained from living patients.

A total of 15 SEB, 15 SCC, and 17 BCC samples (i.e., 47 tumors from 47 patients) met these criteria and were included in the final dataset. The number of mitoses per 10 randomly selected ×400 high-power fields (HPF, with a field diameter of 400 μm) were determined by a pathologist (GS), blinded to clinical data. The study was approved by the Swedish Ethical Review Authority (reference number 2020–02835) and was conducted in accordance with the principles outlined in the Declaration of Helsinki. Written informed consent was obtained from all living patients.

### Immunohistochemistry and digital image analysis

**Fig. 1:**
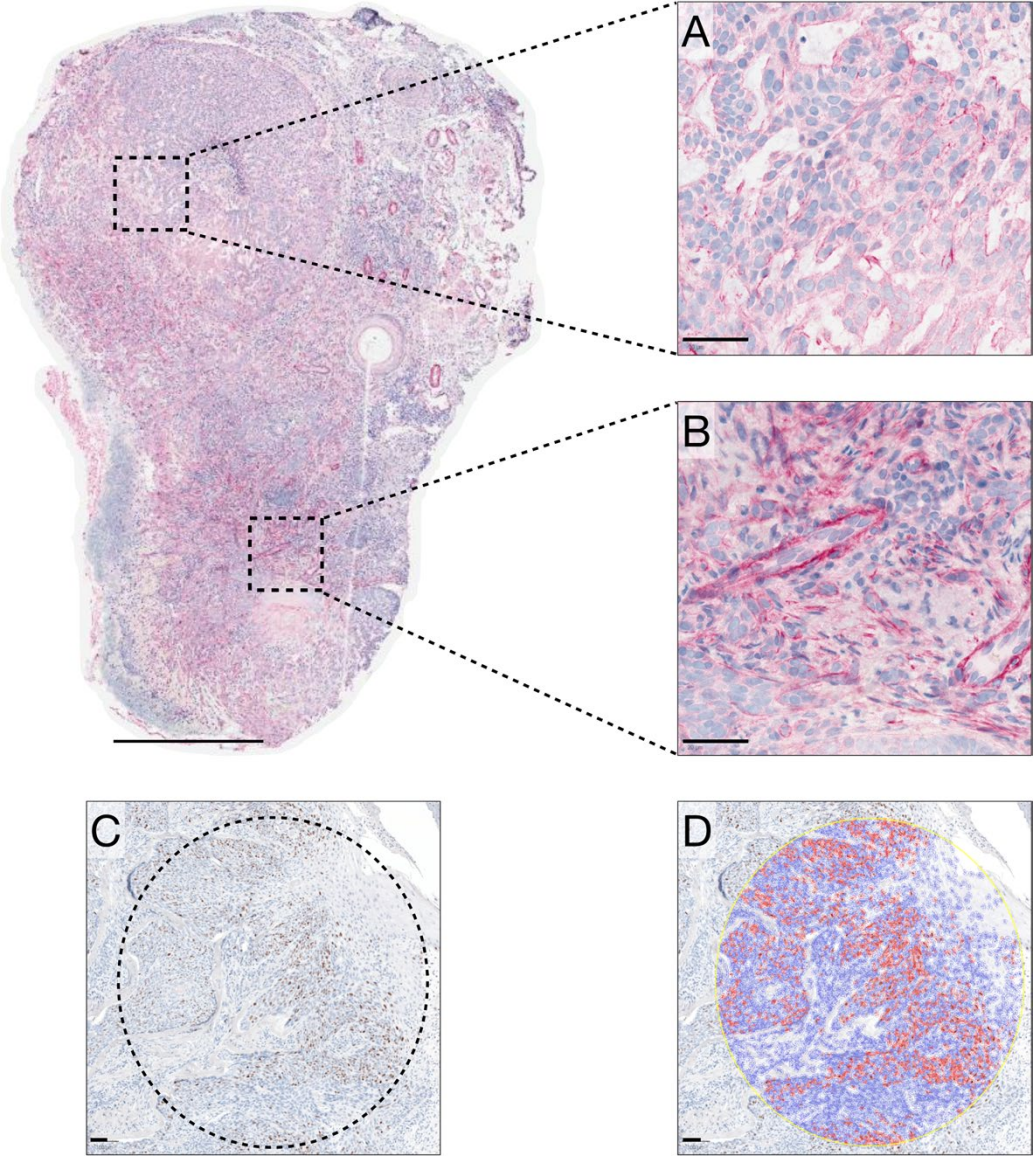
Example of sebaceous carcinoma of the eyelid stained immunohistochemically for (A, B) Smoothened (SMO; red chromogen) and (C, D) the proliferation marker Ki67 (brown chromogen). Expression levels of both markers were quantified by digital image analysis. SMO expression is evident in the (**A**) nodular portion near the eyelid margin, and in (**B**) the infiltrative area toward the eyelid base. (**C**) Proliferative activity was assessed in circular hot-spot regions 1.13 mm in diameter (4 mm^2^) that contained ≥ 200 tumor cells; multiple regions were examined, and the one with the highest Ki67-positive-to-total-cell ratio was selected. (**D**) Digital overlay of the hot spot, with Ki67-positive cells pseudo-colored red and Ki67-negative cells blue. Scale bars: main images, 0.5 mm; insets, 50 μm.

Formalin-fixed, paraffin-embedded tumor sections were stained for SMO using rabbit anti-human polyclonal antibodies (LifeSpan BioSciences Inc., Shirley, MA, USA) with red chromogen on a Leica Bond-III automated stainer (Leica, Wetzlar, Germany). A pathologist (GS) determined the optimal dilution (1:400) by testing concentrations from 1:50 to 1:800. Ki67 staining used rabbit monoclonal antibodies (Cell Signaling Technology, Danvers, MA, USA) with brown chromogen at 1:600 dilution. After staining, the slides were digitally scanned at ×400 magnification (Ocus 40, Grundium Oy, Tampere, Finland, Fig. 1). Digital image analysis was performed using QuPath Bioimage analysis software (v0.2.3)^16^. SMO expression was quantified by marking the entire region of the slide containing invasive tumor tissue. Since SMO is a transmembrane protein, we measured the mean optical density (OD) of the antibody signal within the cytoplasm of cells in the marked area, as described in previous studies^17,18^. Ki67 expression was quantified by selecting the circular 1.13 mm-diameter area (corresponding to 4 mm^2^) containing at least 200 tumor cells that exhibited the highest ratio of Ki67-positive to total tumor cells, as described previously^19^. Positive staining was defined as any brown chromogen above background.

### Statistical analyses

To compare continuous variables across more than two groups, the Kruskal-Wallis test was used, followed by post hoc pairwise comparisons using Dunn’s test. In Dunn’s test, *P* values were adjusted upward by multiplying them by the number of statistical comparisons to control for family-wise error rates. For other analyses, such as linear regression and the Mann-Whitney *U* test, the significance threshold was adjusted using the Bonferroni correction (0.05 divided by 5), to ensure a conservative approach. All *P* values were two-sided. Associations between mitotic counts and SMO expression were assessed using linear regression analysis. Statistical analyses were performed using GraphPad Prism, version 10.2.3 (GraphPad Software, LLC, La Jolla, CA, USA).

## Results

### Descriptive statistics

The mean age at diagnosis for the 47 included patients was 84 years (standard deviation [SD] 10), with 22 (47%) patients being male and 25 (53%) being female. All included BCC were non-nodular. Of the 15 SEB cases, 6 (40%) exhibited intraepithelial growth extending beyond the lateral borders of the areas with invasive tumor growth (pagetoid spread). An overview of the histopathological characteristics and subtypes of the 47 tumors is presented in Table 1.

**Table 1:** Histopathological characteristics of 47 tumors from 47 patients.

Tumor type	*n*	Key Features
Basal cell carcinoma (BCC)	17	EADO stage, n*
		-IIB: 10
		-IIIB: 7
		No patients in stages I, IIA, IIIA, IIIC, or IV
Squamous cell carcinoma (SCC)	15	Differentiation, *n*
		-Well differentiated: 4
		-Moderately differentiated: 9
		-Poorly differentiated: 2
		AJCC 8th Edition T-category, *n*^†^
		-T1a: 5
		-T1b: 6
		-T2a: 3
		-T3c: 1
Sebaceous carcinoma (SEB)	15	Differentiation, *n*
		-Poorly differentiated: 6
		-Moderately differentiated: 9
		Pagetoid spread
		-Present in 6/15 (40%)
		WHO Grade, *n*^‡^
		-Grade I: 8
		-Grade II: 3
		-Grade III: 4
		Growth patterns, *n*
		-Lobular: 11
		-Comedocarcinoma: 1
		-Papillary: 0
		-Mixed: 3

*According to the European Association for Dermato-Oncology (EADO) proposal for an operational staging system adapted to basal cell carcinomas^20^. ^†^According to the American Joint Committee on Cancer (AJCC) staging system for eyelid carcinomas (8th Edition)^21^. ^‡^The WHO grading system for sebaceous carcinoma ranges from well-demarcated tumors (Grade I) to highly invasive growth patterns (Grade III)^22^.

### SMO expression across tumor types

**Fig. 2:**
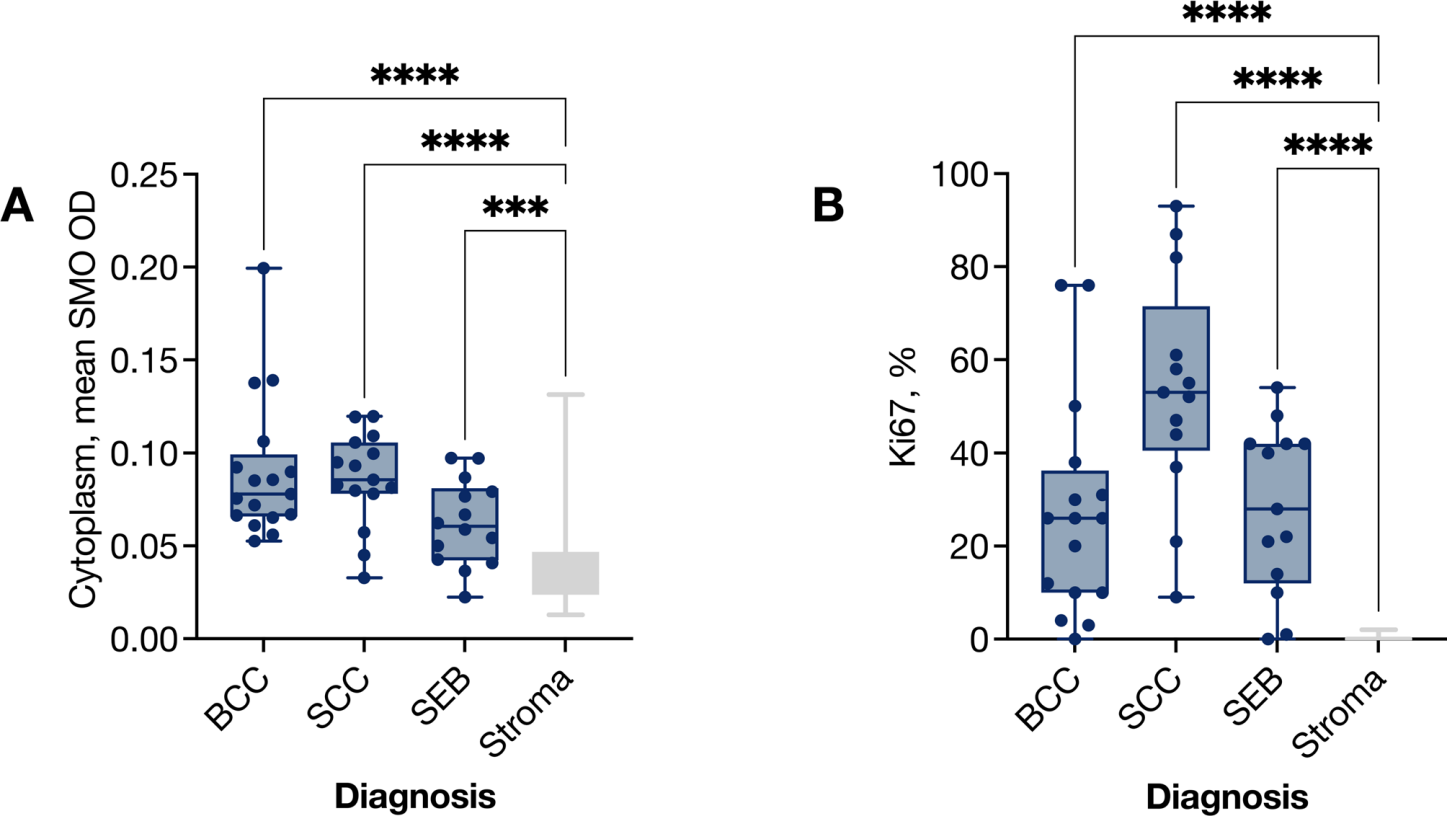
Smoothened (SMO) and Ki67 expression in periocular basal cell carcinoma (BCC), squamous cell carcinoma (SCC), and sebaceous carcinoma (SEB). (**A**) SMO staining intensity was higher in all three tumor types than in the surrounding stroma (> 1 mm from the invasive front), but the values did not differ significantly between tumor types (Kruskal–Wallis, *P* = 0.03; Bonferroni-corrected significance threshold, *P* < 0.01). Post-hoc Dunn tests gave *P* > 0.99 for BCC vs. SCC and *P* = 0.08 for BCC vs. SEB. (**B**) The Ki67 index, quantified digitally in hot-spot areas with the highest proportion of Ki67-positive tumor nuclei, was likewise higher in all tumor types than in stroma, but showed no significant inter-tumor differences (Kruskal–Wallis, *P* > 0.39).

The mean SMO optical density (OD) was 0.09 (SD 0.04) in BCC, 0.09 (SD 0.03) in SCC, and 0.06 (SD 0.02) in SEB. All tumor types had significantly higher SMO expression than non-inflamed stroma (Kruskal-Wallis test, *P* < 0.001, with post hoc pairwise comparisons using Dunn’s test *P* < 0.001 for stroma vs. BCC, *P* < 0.001 for stroma vs. SCC, and *P* < 0.001 for stroma vs. SEB). No significant differences in SMO expression were observed between BCC, SCC, or SEB (Kruskal-Wallis test, *P* = 0.03, at the Bonferroni-corrected significance threshold of 0.01). Post hoc pairwise comparisons using Dunn’s test showed *P* > 0.99 for BCC vs. SCC and *P* = 0.08 for BCC vs. SEB (Fig. 2A). Proliferative activity measured by Ki67 likewise showed no significant inter-tumor differences (Fig. 2B).

### SMO expression versus proliferative activity, degree of differentiation, and pagetoid spread

**Fig. 3:**
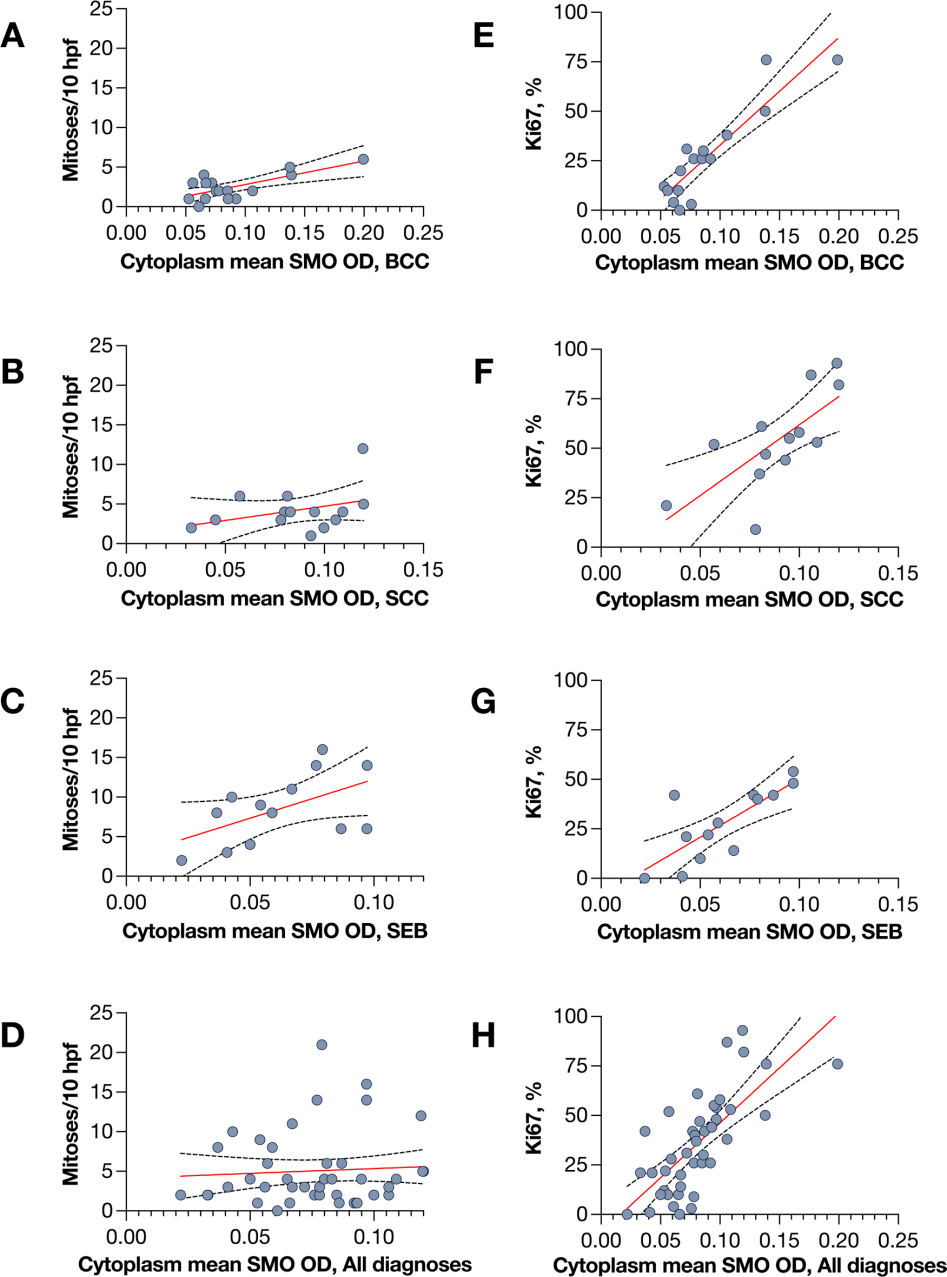
Linear regression analyses of mitotic count and Ki67 proliferation index versus Smoothened (SMO) expression. (A–D) Number of mitoses per 10 high-power fields (HPFs; 400× magnification, field diameter 400 μm) plotted against SMO expression, measured as optical density (OD). (E–H) Ki67 index measured digitally in hot-spot areas with the highest ratio of Ki67-positive cells to total tumor cells, plotted against SMO expression. In basal cell carcinoma (BCC; **A**), SMO expression was significantly associated with mitotic count (R^2^ = 0.50; slope coefficient = 2.97 mitoses per 0.1-OD increase; *P* = 0.002). No significant associations were found at the Bonferroni-corrected significance threshold (*P* < 0.01) in squamous cell carcinoma (SCC; **B**; R^2^ = 0.13; *P* = 0.22), sebaceous carcinoma (SEB; **C**; R^2^ = 0.29; *P* = 0.06), or across all diagnoses combined (**D**; R^2^ = 0.01; *P* = 0.57). By contrast, SMO expression correlated significantly with Ki67 index in BCC (**E**; R^2^ = 0.82; *P* < 0.001), SCC (**F**; R^2^ = 0.52; *P* = 0.005), SEB (**G**; R^2^ = 0.61; *P* = 0.002), and all diagnoses combined (**H**; R^2^ = 0.53; *P* < 0.001).

Linear regression showed a significant association between SMO expression and mitotic count in BCC (R^2^ = 0.50, slope = 2.97 mitoses per 0.1-OD increase, *P* = 0.002) but not in SCC (R^2^ = 0.13, *P* = 0.22), in SEB (R^2^ = 0.29, *P* = 0.06), or in all diagnoses combined (R^2^ = 0.01, *P* = 0.57). In contrast, SMO expression correlated with Ki67 index in every group: BCC (R^2^ = 0.82, *P* < 0.001), SCC (R^2^ = 0.52, *P* = 0.005), SEB (R^2^ = 0.61, *P* = 0.002), and all tumors combined (R^2^ = 0.53, *P* < 0.001, Fig. 3). Ki67 index did not correlate with mitotic count (R^2^ = 0.09, *P* = 0.06).

SMO expression did not differ by degree of differentiation in SCC or SEB (Supplementary Fig. 1), nor between SEB cases with and without pagetoid spread (Supplementary Fig. 2).

## Discussion

This study demonstrates that SMO is expressed in periocular non-nodular BCC, SCC, and SEB, with no significant differences in expression levels among these tumor types. Additionally, higher SMO levels were linked to greater proliferative activity, reflected by a higher Ki67 hot-spot index across all three carcinomas and by a positive correlation with mitotic count in BCC, whereas no such correlation was found in SCC or SEB.

In 2017, Bladen and colleagues analyzed 11 periocular SEB samples and reported higher immunohistochemical expression of Hh pathway proteins—PTCH1, SMO, Gli1, and Gli2—compared to nodular BCC^15^. Our findings revealed similar SMO expression in SEB compared to non-nodular BCC. Although no clinical trials have yet evaluated Hh inhibition in SEB, these comparable expression levels raise the possibility that Hh inhibition could be considered as a therapeutic approach for this aggressive tumor, which is prone to recurrence and metastasis.

Nevertheless, the therapeutic applicability of Hh inhibition in periocular SEB remains uncertain. The interplay between cancer and the immune microenvironment—as well as secondary resistance—may reduce the effectiveness of Hh inhibitors outside the context of BCC^23–26^. This underscores the need for future studies with larger cohorts and diverse anatomic sites to clarify these issues, as well as the potential role for combination therapies or agents targeting downstream pathway components. If Hh inhibition is employed in the neoadjuvant setting to decrease tumor size and extent before surgery, the risk of resistance or refractory tumor growth may be less critical.

Recent studies have also investigated newer Hedgehog inhibitors, such as patidegib, an SMO inhibitor with both systemic and topical formulations^23^. In BCC, topical patidegib 2% gel has shown efficacy while reducing some adverse effects associated with vismodegib and sonidegib. Although patidegib is not yet used for SEB, its mechanism of action—targeting SMO—could theoretically extend to other tumors expressing this pathway component.

In our study, SMO expression in SEB was comparable to that of non-nodular BCC, raising the possibility that SEB might also respond to Hh inhibition. However, the lack of a linear correlation with mitotic activity in SEB as well as for SCC, in contrast to BCC; might indicate that SEB diverges from BCC in terms of treatment response to Hh inhibition.

We also propose that Hh inhibitors be tested in future clinical trials for SEB, especially in cases with significant tumor size, intraepithelial or perineural growth, and extensive anatomic involvement. Co-expression of biomarkers such as CD56 or CXCR4 has been shown to predict non-response to Hh inhibition in certain cancers^27^. It is possible that similar predictors of therapeutic response exist in SCC and SEB. Identifying such biomarkers would help stratify patients, determining who is most likely to benefit from Hh inhibitors and who may not respond to the treatment. Additionally, protein expression in metastases has been shown to differ from that in primary tumors in other cancer types^28^. Therefore, confirming SMO expression in both the primary tumor and any metastases might be beneficial when considering Hh inhibition as a treatment strategy for SEB and SCC.

The association between higher SMO expression and increased mitotic activity in BCC is notable, as it may correlate with more aggressive tumor variants. This could help explain the efficacy of Hh inhibition in advanced and metastatic BCC. It also highlights a key difference between our study, which focused on non-nodular BCC (typically more aggressive and with higher mitotic counts), and the previous study, which identified greater SMO expression in SEB than in nodular BCC^15^. Thus, while SMO expression might be higher in SEB compared to nodular BCC, it appears similar when comparing SEB to more aggressive subtypes of BCC that infiltrate surrounding tissues with narrower cords or strands of cells.

### Limitations

This study has several limitations. First, we included relatively small cohorts of patients with SCC, BCC, and SEB, which may not be fully representative of all patients with these diagnoses. Larger cohorts would likely yield more generalizable results and allow for comparisons of expression levels between different subtypes of BCC, SCC, and SEB.

Second, while the Hedgehog pathway involves several key proteins—such as PTCH1, SMO, Gli1, and Gli2—we only examined SMO. Including additional pathway components could have offered a more comprehensive understanding of Hh pathway activity in these tumors.

Third, we assessed protein expression levels through immunohistochemistry but did not analyze RNA or other molecular levels, which may provide additional insights into pathway regulation.

Fourth, the strong correlation between SMO and Ki67 may be partly technical: tissues with suboptimal fixation (e.g., prolonged cold ischemia, over-fixation, or older blocks) often show uniformly reduced antigenicity, producing artifactual covariation between antibodies. The discrepancy between manual mitotic counts and digitally quantified Ki67 highlights methodological differences, but prior studies indicate that automated Ki67 analysis is more reproducible and prognostically informative than manual mitotic scoring^19,29,30^.

## Conclusions

Our findings demonstrate similar levels of SMO expression in periocular non-nodular BCC, SEB, and SCC. These results suggest that clinical trials should be conducted to evaluate the efficacy of Hh inhibition for SEB and SCC, as it has been shown to be effective in BCC.

## Electronic supplementary material

Below is the link to the electronic supplementary material.

Supplementary Material 1

Supplementary Material 2

## Data Availability

The raw data for this study is available from the corresponding author upon reasonable request.
