# Supplementary figures and images for: Immunohistochemical expression of smoothened in periocular basal cell, squamous cell and sebaceous carcinomas
Source: Sci Rep. 2025 Jun 20;15:20182. doi: 10.1038/s41598-025-06011-y (PMC12181246; doi:10.1038/s41598-025-06011-y)

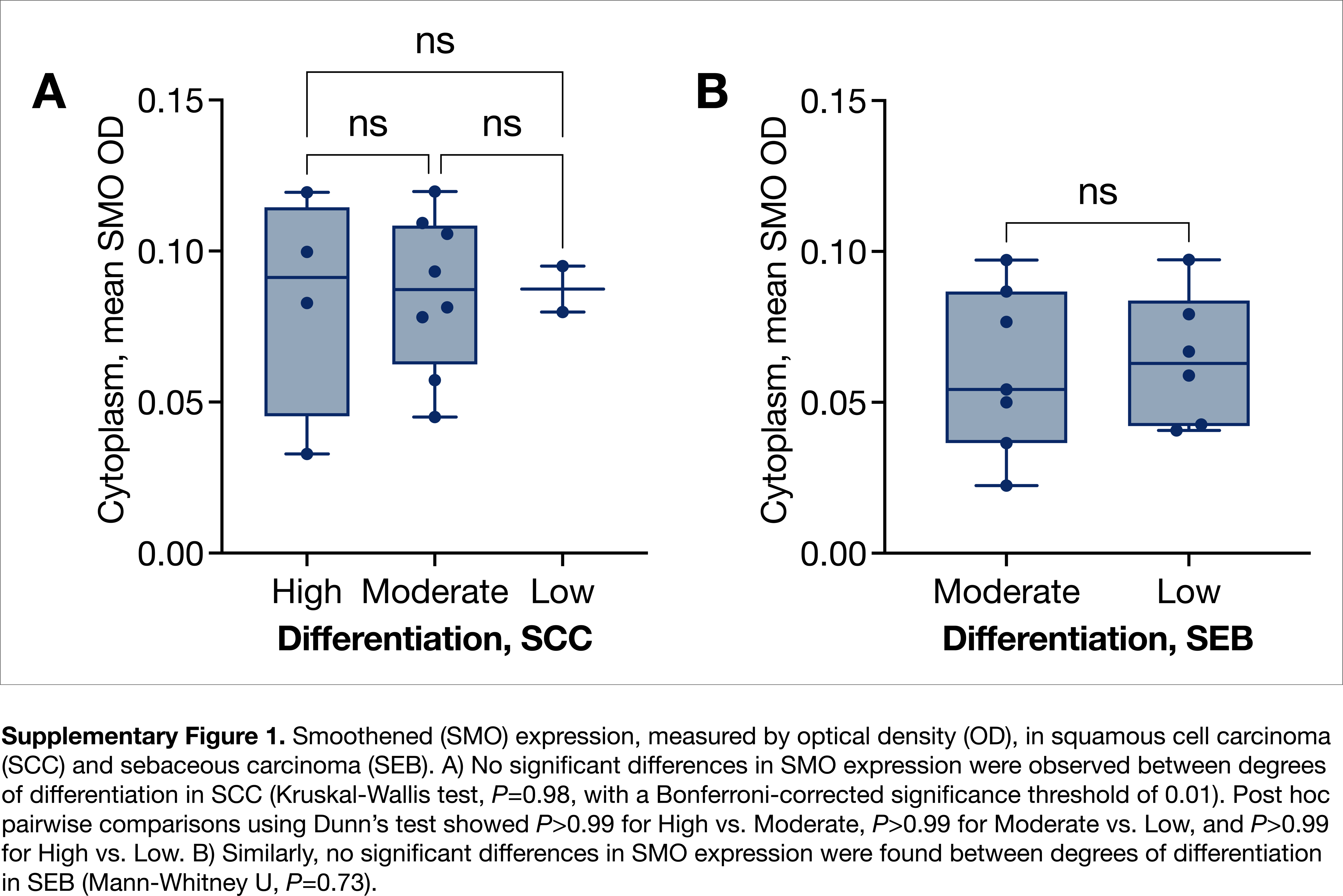

Supplement: Supplementary file 1 — Supplementary Material 1 [file 41598_2025_6011_MOESM1_ESM.tiff]

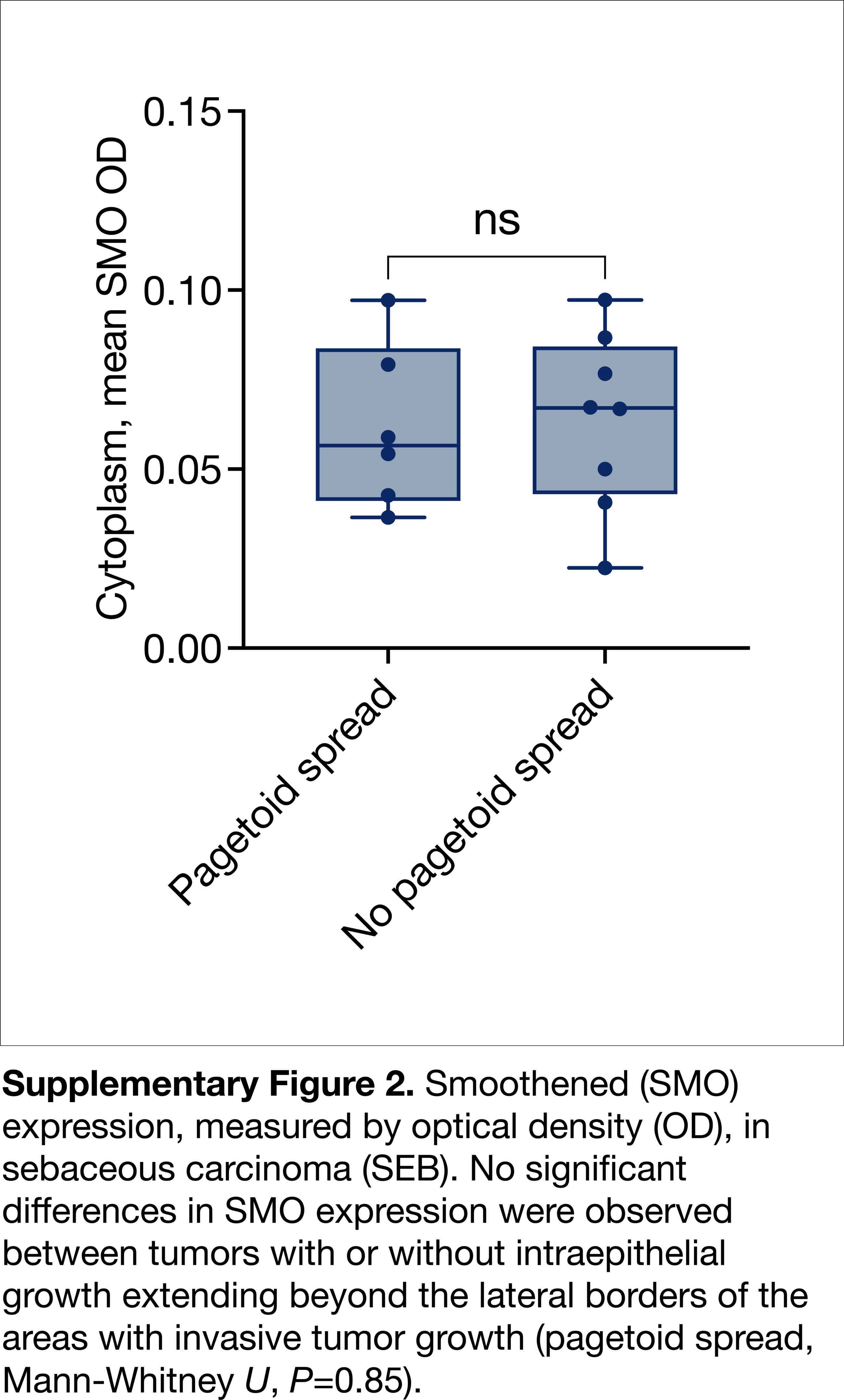

Supplement: Supplementary file 2 — Supplementary Material 2 [file 41598_2025_6011_MOESM2_ESM.tiff]
